# Bayesian Inference of Hidden Cognitive Performance and Arousal States in Presence of Music

**DOI:** 10.1109/OJEMB.2024.3377923

**Published:** 2024-03-18

**Authors:** Saman Khazaei, Md Rafiul Amin, Maryam Tahir, Rose T. Faghih

**Affiliations:** Department of Biomedical EngineeringNew York University 5894 New York NY 10010 USA; Department of Electrical and Computer EngineeringUniversity of Houston 14743 Houston TX 77004 USA

**Keywords:** Affective computing, biomedical signal processing, estimation, state-space methods

## Abstract

*Goal:* Poor arousal management may lead to reduced cognitive performance. Specifying a model and decoder to infer the cognitive arousal and performance contributes to arousal regulation via non-invasive actuators such as music. *Methods:* We employ a Bayesian filtering approach within an expectation-maximization framework to track the hidden states during the $n$-back task in the presence of calming and exciting music. We decode the arousal and performance states from the skin conductance and behavioral signals, respectively. We derive an arousal-performance model based on the Yerkes—Dodson law. We design a performance-based arousal decoder by considering the corresponding performance and skin conductance as the observation. *Results:* The quantified arousal and performance are presented. The existence of Yerkes—Dodson law can be interpreted from the arousal-performance relationship. Findings display higher matrices of performance within the exciting music. *Conclusions:* The performance-based arousal decoder has a better agreement with the Yerkes—Dodson law. Our study can be implemented in designing non-invasive closed-loop systems.

## I. Introduction

The word cognition refers to “the mental action of acquiring knowledge and understanding through thought and experience”, which emphasizes the dynamics of learning as opposed to the participant's previous knowledge [3]. Human emotional status directly impacts the cognition [4]. Particularly, arousal, which refers to an intensity level of human emotions, can determine cognitive performance in performing a cognitive task [5]. Cognitive tasks are those that require a person to mentally process new information, retrieve that information from memory, and use it at a later time [6]. The term cognitive performance describes the overall performance of the cognitive functions over a cognitive task. Human cognitive functions are diverse and can be divided into two main branches, namely, basic functions and higher-level cognitive functions. Basic cognitive functions include attention, working memory (WM), and perception, while higher-level cognitive functions consist of speech and language, decision-making, and executive control [7]. In this research, we investigate the underlying arousal and performance state during the $n$-back task –which requires WM usage– in the presence of music.

WM is a basic cognitive function that enables the temporary storage and manipulation of information [8]. While working memory, by itself, is a basic cognitive function, it would serve as a core component of higher cognitive function, and several cognitive tasks involve working memory usage [9]. Here, the $n$-back task serves as a cognitive task of interest. The $n$-back task mainly executes the working memory. Additionally, there is evidence of executive control and attention involvement when performing the $n$-back task [10]. A single block of an $n$-back task includes sets of letters known as the stimulus. For each stimulus, a participant is supposed to realize whether the presented stimulus matches the $n$th previous one or not. The higher order of “$n$” would result in a higher cognitive load of WM [11]. The term cognitive load implies the occupied WM resources [12].

Human emotion has been modeled using different approaches. In one of the early and well-known models, emotion has been demonstrated by Ekman and Friesen using 6 distinct categories: happiness, sadness, surprise, fear, anger, and disgust. However, there was a lack of continuity in that paradigm [13], [14]. A more advanced model has been developed by Russell such that emotion can be recognized continuously using two orthogonal axes—valence and arousal. The term arousal denotes the intensity level of emotion associated with the sympathetic nervous system, and valence has been related to the pleasantness and unpleasantness of the emotion [5], [14]. The electrodermal activity (EDA) can be considered as an informative index of arousal [15]: The human autonomic nervous system is composed of three main branches, namely, the sympathetic nervous system, the parasympathetic nervous system, and the enteric nervous system [16]. Sympathetic nerve fibers are responsible for the innervation of sweat glands [17]. The variations in sweat secretions can be measured from the skin. Therefore, the skin conductance signal (a measure of EDA) can be applied as a metric to monitor the arousal level [18].

The arousal and working memory association can depend on multiple factors, such as underlying neurotransmitter production, valence, working memory tasks, and personal characteristics [9]. Based on the observed association between norepinephrine – a type of neurotransmitter that is positively correlated with arousal – and performance in working memory tasks, it has been hypothesized that moderate levels of arousal can improve working memory, while extreme levels of arousal may impair working memory [19]. This hypothesis complies with the Yerkes—Dodson law. The Yerkes—Dodson law – known as an inverted-U law in psychology– explains that an extremely low arousal level can lead to a lack of attention while extremely high arousal may result in a distraction in which both cases would prevent reaching the optimal cognitive performance [20]. To support this hypothesis, one may exclusively investigate the $n$-back task as a working memory task of interest. Specifically, to perform the $n$-back task, the focus of attention serves as an essential factor. The focus of attention is mainly provided by attentional capacity, which is the limited capacity system. Optimal arousal can result in high attentional capacity, while excessively high and low arousal levels lead to reductions in attentional capacity, which follows the Yerkes—Dodson law [21], [22].

The inverted-U law offers us an opportunity to regulate arousal such that it boosts cognitive performance. External non-invasive interventions such as background music can be employed to influence the mood or arousal level of an individual [23]. Particularly, the type of music can be an effective factor in cognitive performance regulation and designing non-invasive arousal actuators. Previous studies reveal that rock music may reduce productivity in performing the $n$-back task, while no music or listening to country or jazz music can enhance the participant's performance in the course of the $n$-back task [24]. Given the non-invasiveness, accessibility, daily music listening time, and advances in music streaming platforms, the idea of using music to impact cognitive states seems to be far-reaching and worthy of consideration. Hence, we employ the collected behavioral data and skin conductance signal throughout the $n$-back tasks in the presence of two types of music selected by the participants [25]. The music component was used to mock the low and high arousing environment that can possibly affect the performance. Hence, the participants were asked to provide music with calming and exciting content, which are mainly different in terms of arousal rather than valence [5].

The cognitive performance and arousal states are often presented as discrete measurements, such as discrete ratings provided by either subjects or observers. The discrete measurement prevents us from continuous tracking of the arousal and performance. Using the Bayesian filtering approach within an expectation-maximization framework, we decode the continuous performance and arousal state [14], [26].

The objectives of this study can be listed as decoding the arousal and performance in the presence of music, presenting performance indices within each music session as well as task difficulty, evaluating the arousal-performance link, and developing a performance-based arousal decoder accordingly. To obtain the sparse autonomic nervous system (ANS) activation from the skin conductance, we perform a signal deconvolution [27]. To estimate the arousal state from the recovered ANS activations, we use a marked point process (MPP) filtering. To estimate the latent cognitive performance state, we employ the sequence of correct/incorrect responses and the reaction time at each trial [28], [29]. In order to estimate performance-based arousal, we consider the combination of skin conductance data and cognitive performance as the effective observation. Particularly, we utilize the arousal-related events derived from ANS activation and the continuous performance to form the observation vector. Thereafter, we decode the arousal using the proposed Bayesian decoder.

## II. Materials and Methods

### A. Dataset

**Fig. 1.**
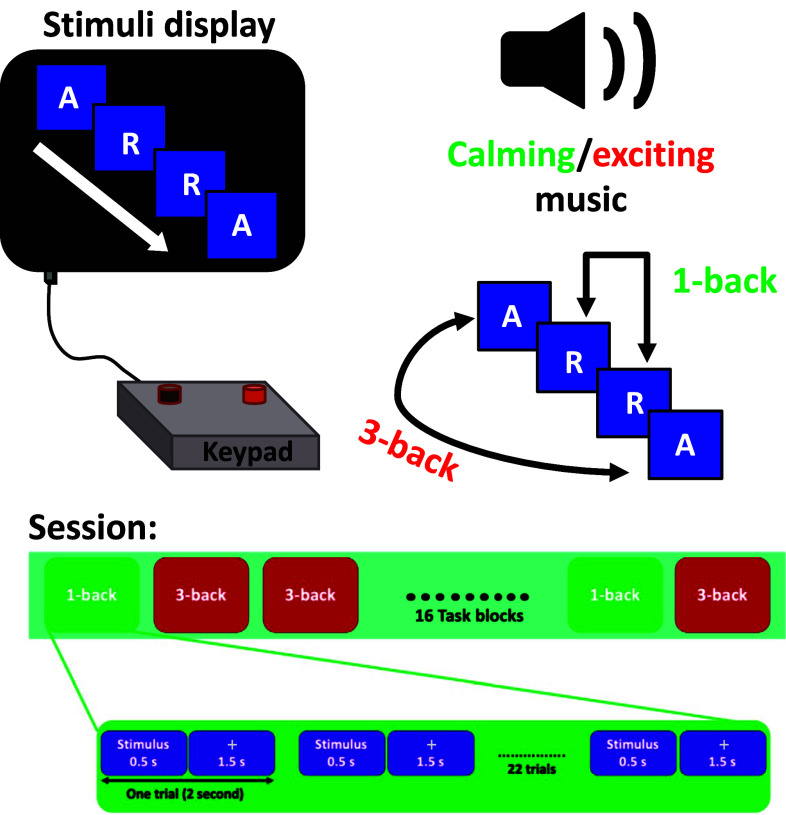
Summary of the experimental setup ($n$-back task). The 1-back and 3-back task blocks were implemented within two sessions in the presence of calming and exciting background music.

The experimental data used in this research was collected under the approval of the Institutional Review Board at the University of Houston, Houston, Texas, USA. The experiment was originally conducted to investigate the viability of applying music as a neurofeedback mechanism in the course of n-back experiment [25]. Several behavioral data and physiological signals were recorded from 6 novice participants during the $n$-back task in the presence of two sessions of calming and exciting background music. The participants were asked to select the music with calming and exciting content. The music was applied to simulate the low and high arousing environment. Hence, the selected content of music was supposed to be different in terms of arousal rather than valence. According to Russel's emotion model, while calming music is pleasant and minimally arousing, music with an exciting subject is pleasant and highly arousing [5]. The calming background music was played in the first session, and the exciting background music was presented during the second session. To avoid fatigue and minimize learning impact on behavioral measurements, equal numbers of 1-back and 3-back task blocks were randomly distributed within two sessions of calming and exciting background music, and instructions were provided at the beginning of each trial. A total number of 32 task blocks were implemented (16 task blocks within each session). Each task block was initialized with the 5 seconds instruction period, followed by 22 trials, with 0.5 seconds for displaying the letter. In addition to 0.5 seconds display time, the participant had 1.5 seconds to deliver the response and press a Chronos Keypad button to determine whether the presented letter was the same as the the $n$th previous letter (pressing the target button) or it was mismatched (pressing non-target button). Therefore, the total stimulus time at each task block was 49 seconds. In total, a participant performed 704 trials (i.e., 2 sessions × 16 task blocks × 22 trials). At the end of each task, a 10 seconds relaxation segment was contrived. After 8 blocks (halfway mark for each session), a 20 seconds relaxation section was implemented, and between the sessions, there was a 2-minute relaxation break. During the data collection process, the participants were asked to engage in the task and avoid unnecessary movements. The only required movement was related to pressing either the target button or the non-target button on the Chronos Keypad. The recorded behavioral data comprised of reaction time and sequence of correct/incorrect responses. A summary of the employed cognitive task is presented in Fig. 1.

### B. Inference of Brain Activation From Skin Conductance Measurements

In order to infer the neural impulse train from the raw skin conductance signal, an appropriate deconvolution method needs to be employed. Applying a coordinate descent approach we recover the sparse arousal events due to ANS activation [30], [31]. A detailed description of the approach can be found in the supplementary materials.

### C. A Marked Point Process State-Space Model for Arousal

Similar to [14], we assume a random walk model for the hidden arousal state $\hat{x}_{j}$ such that

\begin{align*}
\hat{x}_{j} = \hat{x}_{j-1} + \epsilon _{j}, \tag{1}
\end{align*}

where $\epsilon _{j} \sim \mathcal {N} \;(0,\sigma ^{2}_{\epsilon })$ is the process noise and $j$ stands for the time index. Following the marked point process filtering approach in [14], we consider Bernoulli distribution for the arousal events $n_{j}$, with probability mass function $a_{j}^{n_{j}}(1 - a_{j})^{1 - n_{j}}$ such that $P(n_{j} = 1) = a_{j}$. We relate $\hat{x}_{j}$ to $a_{j}$ by applying a sigmoid transform similar to [26]. Thus,

\begin{align*}
a_{j} = \frac{1}{1 + e^{-(\hat{x}_{j} + \beta)}}, \tag{2}
\end{align*}

where $\beta$ is a constant that can be derived from $\scriptsize {\beta \approx \log (\frac{a_{0}}{1 - a_{0}})}$ and $a_{0}$ is the average probability of observing an impulse during the experiment. Similar to [14], continuous-valued amplitude $r_{j}$ of each neural impulse may be represented as

\begin{align*}
r_{j} = \hat{\gamma }_{0} + \hat{\gamma }_{1}\hat{x}_{j} + v_{j}, \tag{3}
\end{align*}

where $r_{j}$ is the amplitude of the observed impulse, $v_{j}\sim \mathcal {N} \,(0,\sigma ^{2}_{v})$ presents the sensor noise, $\hat{\gamma }_{0}$ and $\hat{\gamma }_{1}$ are the unknown parameters to be determined. Consequently, the joint density function for the observed neural stimuli is

\begin{align*}
p(n_{j} \cap r_{j}|\hat{x}_{j})= \left\lbrace \begin{array}{ll} 1-a_{j} \qquad \qquad \qquad & \text{if} \; n_{j} = 0\\
a_{j}\frac{1}{\sqrt{2\pi \sigma _{v}^{2}}}e^{\frac{-(r_{j}-\hat{\gamma }_{0}-\hat{\gamma }_{1}\hat{x}_{j})^{2}}{2\sigma _{v}^{2}}} & \text{if}\; n_{j} = 1 \end{array}\right.. \tag{4}
\end{align*}

The derivation of a marked point process state-space decoder for arousal is described in the supplementary materials.

### D. A State-Space Model for Performance

Inspired by the proposed state-space model in [28], we consider an autoregressive model for the cognitive performance state.

\begin{align*}
z_{k} = \rho z_{k-1} + w_{k}, \tag{5}
\end{align*}

where $z_{k}$ is a hidden performance state, $w_{k}\sim \mathcal {N} \,(0,\sigma ^{2}_{w})$ stands for the process noise, $\rho$ is the unknown coefficient, and $k$ is the trial number during the experiment.

Similar to [28], we form the observation model by specifying one binary observation (correct/incorrect response at $k^{th}$ trial) and one continuous observation (reaction time of the corresponding response). The Bernoulli probability model is assumed for the binary responses with the probability mass function of $p_{k}^{m_{k}}(1 - p_{k})^{1 - m_{k}}$. Applying sigmoid transform we may express the $p_{k}$ in terms of $z_{k}$ such that

\begin{align*}
p_{k} = \frac{1}{1 + e^{-(z_{k} + \mu)}}. \tag{6}
\end{align*}

The constant term $\mu$ can be determined from $\scriptsize {\mu \approx \log (\frac{p_{0}}{1 - p_{0}})}$ where $p_{0}$ is the average probability of having a correct response.

The reaction time $\tau _{k}$ can be related to the performance state using:

\begin{align*}
l_{k} = \log (\tau _{k}) = \alpha _{0} + \alpha _{1} z_{k} + \delta _{k}, \tag{7}
\end{align*}

where $\delta _{k}\sim \mathcal {N}\; (0,\sigma ^{2}_{\delta })$, and $l_{k}$ is the $\log$ of reaction time at each trial.

The performance state decoder's equations can be found in the supplementary materials.

### E. Performance as a Function of Arousal

By utilizing both decoded arousal ($\hat{x}_{j}$) and performance states ($z_{k}$), we define an arousal-performance function inspired by the inverted-U law [1], [20]:

\begin{align*}
Y_{k} = \hat{\lambda }_{1} X_{k}^{2} + \hat{\lambda }_{2} X_{k} + \hat{\lambda }_{3} + e_{k}, \tag{8}
\end{align*}

where $Y_{k}$ presents the standard score of the performance state at each n-back trial and $X_{k}$ stands for the standard score of the average arousal—derived from marked point process filter—at each n-back trial. Thus, the observed data points consist of ($X,Y$); $e_{k}$ is assumed to follow a white noise structure, $e_{k}\sim \mathcal {N}\,(0,\sigma ^{2}_{e})$ and, $\hat{\lambda }_{1}$, $\hat{\lambda }_{2}$, and $\hat{\lambda }_{3}$ are the unknown parameters that can be determined by robust fitting with bisquare weighting. It is not advised to use the ordinary least-squares method since the data points here consist of different layers from multiple different trials. Instead, a robust fitting with bisquare weights can be employed using a MATLAB function (*fitlm*).

### F. A Performance-Based Arousal State-Space Model

According to Table I, the p-values for the linear term $\hat{\lambda }_{2}$ are high for most of the participants which display that the statistical significance of $\hat{\lambda }_{2}$ is considerably low compared to $\hat{\lambda }_{1}$ and $\hat{\lambda }_{3}$. Based on the inverted quadratic relationship between arousal and performance and by ignoring the linear term $\hat{\lambda }_{2}$, we may modify the arousal state observation model based on the performance. Considering the arousal level at each trial, the state follows the previous random walk model such that

\begin{align*}
\tilde{x}_{k} &= \tilde{x}_{k - 1} + u_{k}, \tag{9}
\end{align*}

where $\tilde{x}_{k}$ stands for the performance-based arousal at $k^{th}$ trial and $u_{k} \sim \mathcal {N}\,(0,\sigma ^{2}_{u})$ is the process noise. Similar to the proposed marked point process approach, we specify a Bernoulli distribution for $\tilde{n}_{k}$ at each trial where $\tilde{n}_{k}$ stands for the arousal events at each trials (average of neural impulses over each trial). Note that each trial takes 2 seconds and we might have more than one impulse at each trial; however, it does not affect our arousal events' vector $\tilde{N} = \lbrace \tilde{n}_{1}, \tilde{n}_{2},{\ldots },\tilde{n}_{k}\rbrace$ since the hidden state is defined based on trials. Hence, $\tilde{n}_{k}$ only takes 0 or 1 to indicate the arousal events in particular period. We relate the state $\tilde{x}_{k}$ to the probability of arousal events occurrence $\phi$ at trial $k$ as before

\begin{align*}
\phi _{k} &= \frac{1}{1 + e^{-(\tilde{x}_{k} + q_{0})}}, \tag{10}
\end{align*}

where $q_{0}$ is a constant that can be derived from $\scriptsize {q_{0} \approx \log (\frac{\phi _{0}}{1 - \phi _{0}})}$ and $\phi _{0}$ is the average probability of having the arousal event.

**TABLE I:** Arousal-Performance Regression Model Results

p-value			
Participant	$\hat{\lambda }_{1}$	$\hat{\lambda }_{2}$	$\hat{\lambda }_{3}$
1	3.14e-05	0.0197	0.0048
2	4.13e-09	2.04e-25	5.71e-06
3	1.20e-07	0.10985	0.0001
4	5.89e-45	0.0594	8.34e-25
5	3.53e-18	0.0110	4.13e-08
6	2.91e-18	0.0455	1.7e-10

Additionally, the observation model contains the continuous value of performance state $z_{k}$ and, continuous-valued arousal event's amplitude $\tilde{r}_{k}$. Hence,

\begin{align*}
z_{k} &= \tilde{\lambda }_{1} \tilde{x}^{2}_{k} + \tilde{\lambda }_{0} + \psi _{k}, \tag{11}
\end{align*}

where $ \tilde{\lambda }_{1}$ and $\tilde{\lambda }_{0}$ are the unknown parameters, and

\begin{align*}
\tilde{r}_{k} &= \tilde{\gamma }_{0} + \tilde{\gamma }_{1}\tilde{x}_{k} + \zeta _{k}, \tag{12}
\end{align*}

where, $\tilde{r}_{k}$ stands for the average value of neural impulses at each trial. Also, $\tilde{\gamma }_{0}$ and, $\tilde{\gamma }_{1}$ are the unknown parameters. Similarly, $\psi _{k} \sim \mathcal {N}\,(0,\sigma ^{2}_{\psi })$ and $\zeta _{k} \sim \mathcal {N}\,(0,\sigma ^{2}_{\zeta })$ are assumed to be Gaussian. The joint density function for the occurred arousal event is

\begin{align*}
\; p(\tilde{n}_{k} \cap \tilde{r}_{k}|\tilde{x}_{k})=\left\lbrace \begin{array}{ll} 1-\phi _{k} \qquad \qquad \qquad & \text{if}\; \tilde{n}_{k} = 0\\
\, \phi _{k}\frac{1}{\sqrt{2\pi \sigma _{\zeta }^{2}}}e^{\frac{-(\tilde{r}_{k}-\tilde{\gamma }_{0}-\tilde{\gamma }_{1} \tilde{x}_{k})^{2}}{2\sigma _{\zeta }^{2}}} &\text{if}\; \tilde{n}_{k} = 1 \end{array}\right.. \tag{13}
\end{align*}

### G. A Performance-Based Arousal Decoder

We derive a decoder based on the performance-based arousal model. The unknown parameters vector would be $\tilde{\theta } = \lbrace \sigma ^{2}_{u}, \tilde{\lambda }_{1}, \tilde{\lambda }_{0},\sigma ^{2}_{\psi },\tilde{\gamma }_{0},\tilde{\gamma }_{1}, \sigma ^{2}_{\zeta }\rbrace$, and we apply the EM algorithm to jointly estimate $\tilde{x}_{k}$ and $\tilde{\theta }$.

#### 1) E-Step

The E-step consists two subsections namely, forward filtering and backward smoothing. Based upon the observation $\tilde{R}^{K} = \lbrace (\tilde{n}_{1},\tilde{r}_{1},z_{1}),{\ldots },(\tilde{n}_{k},\tilde{r}_{k},z_{k})\rbrace$ up to time $K$, E-step equations can be formulated.

Predict:

\begin{align*}
 \tilde{x}_{k|k-1} =& \tilde{x}_{k-1|k-1},\tag{14}\\
 \tilde{\sigma }^{2}_{k|k-1} =& \tilde{\sigma }^{2}_{k-1|k-1} + \tilde{\sigma }^{2}_{u}, \tag{15}
\end{align*}

Update:

if $\tilde{n}_{k} = 0$

\begin{align*}
\tilde{x}_{k|k} =& \tilde{\sigma }^{2}_{k|k-1}\Bigg[(\tilde{n}_{k} - \phi _{k|k}) \tag{16}\\
 & +\frac{\left(2\tilde{\lambda }_{1}\tilde{x}_{k|k})(z_{k} - \tilde{\lambda }_{0} - \tilde{\lambda }_{1}\tilde{x}^{2}_{k|k}\right)}{\sigma ^{2}_{\psi }}\Bigg] + \tilde{x}_{k|k-1},\\
\tilde{\sigma }^{2}_{k|k} =& \Bigg [\phi _{k|k}(1-\phi _{k|k}) + \frac{1}{\tilde{\sigma }^{2}_{k|k-1}}\\
 &- \frac{1}{\sigma ^{2}_{\psi }} \bigg [2\tilde{\lambda }_{1}(z_{k}-\tilde{\lambda }_{0}-\tilde{\lambda }_{1}\tilde{x}^{2}_{k|k})-(2\tilde{\lambda }_{1}\tilde{x}_{k|k})^{2}\bigg ] \Bigg ]^{-1}.\tag{17}
\end{align*}

if $\tilde{n}_{k} = 1$

\begin{align*}
\tilde{x}_{k|k} =& \tilde{\sigma }^{2}_{k|k-1}\Bigg[(\tilde{n}_{k} - \phi _{k|k})+ \frac{\tilde{\gamma }_{1}(\tilde{r}_{k} - \tilde{\gamma }_{0} - \tilde{\gamma }_{1}\tilde{x}_{k|k})}{\sigma ^{2}_{\zeta }}\tag{18}\\
 & \qquad \quad+ \frac{\left(2\tilde{\lambda }_{1}\tilde{x}_{k|k})(z_{k} - \tilde{\lambda }_{0} - \tilde{\lambda }_{1}\tilde{x}^{2}_{k|k}\right)}{\sigma ^{2}_{\psi }}\Bigg] + \tilde{x}_{k|k-1},\\
\tilde{\sigma }^{2}_{k|k} =& \Bigg [\phi _{k|k}(1-\phi _{k|k}) + \frac{1}{\tilde{\sigma }^{2}_{k|k-1}} + \frac{\tilde{\gamma }^{2}_{1}}{\sigma ^{2}_{\zeta }}\\
 & - \frac{1}{\sigma ^{2}_{\psi }} \bigg [2\tilde{\lambda }_{1}(z_{k}-\tilde{\lambda }_{0}-\tilde{\lambda }_{1}\tilde{x}^{2}_{k|k})-(2\tilde{\lambda }_{1}\tilde{x}_{k|k})^{2}\bigg ] \Bigg ]^{-1}.\tag{19}
\end{align*}

In order to solve for $\tilde{x}_{k|k}$, we have utilized a MATLAB function called *fzero* which solves for the roots of non-linear equations.

In order to acquire the smoothed state $\tilde{x}_{k|K}$ and smoothed variance $\tilde{\sigma ^{2}}_{k|K}$, we reverse the direction:

\begin{align*}
\tilde{A}_{k} &= \frac{\tilde{\sigma }^{2}_{k|K}}{\tilde{\sigma }^{2}_{k+1|k}},\tag{20}\\
\tilde{x}_{k|K} &= \tilde{x}_{k|k}+ \tilde{A}_{k}(\tilde{x}_{k+1|K}-\tilde{x}_{k+1|k}),\tag{21}\\
\tilde{\sigma }^{2}_{k|K} &= \tilde{\sigma }^{2}_{k|k}+\tilde{A}^{2}_{k}(\tilde{\sigma }^{2}_{k+1|K}-\tilde{\sigma }^{2}_{k+1|k}). \tag{22}
\end{align*}

By utilizing the proposed approach in [14] and [26], we derive the expected values of $\tilde{x}_{k}^{2}$, and $\tilde{x}_{k}\tilde{x}_{k-1}$ as

\begin{align*}
\mathbb {E}[\tilde{x}_{k}^{2}] &= \tilde{x}_{k|K}^{2} + \tilde{\sigma }^{2}_{k|K},\tag{23}\\
\mathbb {E}[\tilde{x}_{k+1}\tilde{x}_{k}] &= \tilde{x}_{k+1|K}\tilde{x}_{k|K} + \tilde{A}_{k}\tilde{\sigma }^{2}_{k+1|K}. \tag{24}
\end{align*}

Since we consider a quadratic function to relate the arousal to performance, we first derive an expression for $\mathbb {E}[\tilde{x}_{k}^{4}]$.

\begin{align*}
\mathbb {E}[\tilde{x}_{k}^{4}] &= \mathbb {E}[(\tilde{x}_{k}^{2})^{2}] \\
 &= \left(\mathbb {E}[\tilde{x}_{k}^{2}]\right)^{2} + var(\tilde{x}_{k}^{2}).\tag{25}
\end{align*}

For approximating the last term ($var(\tilde{x}_{k}^{2})$) in (25), similar to [32], we employ the second order Taylor series such that

\begin{align*}
var(\tilde{x}_{k}^{2}) \approx 4 \left(\mathbb {E}[(\tilde{x}_{k})]\right)^{2} \tilde{\sigma }^{2}_{k|k} + 2 \left(\tilde{\sigma }^{2}_{k|k}\right)^{2}. \tag{26}
\end{align*}

Therefore,

\begin{align*}
\mathbb {E}[\tilde{x}_{k}^{4}] \approx \left(\mathbb {E}[\tilde{x}^{2}_{k}]\right)^{2} + 4 \left(\mathbb {E}[(\tilde{x}_{k})]\right)^{2} \tilde{\sigma }^{2}_{k|k} + 2 \left(\tilde{\sigma }^{2}_{k|k}\right)^{2}. \tag{27}
\end{align*}

#### 2) M-Step

We denote the location of the trials where arousal events occurred at $\tilde{K} = \lbrace k|\tilde{n}_{k} = 1\rbrace$. Based on the E-step results, we can form a log-likelihood function $Q_{3}$, and find the unknown parameters $\tilde{\theta } = \lbrace \sigma ^{2}_{u}, \tilde{\lambda }_{1}, \tilde{\lambda }_{0},\sigma ^{2}_{\psi },\tilde{\gamma }_{0},\tilde{\gamma }_{1}, \sigma ^{2}_{\zeta }\rbrace$ such that $\tilde{\theta }$ maximizes $Q_{3}$.

\begin{align*}
Q_{3} =& \sum _{k=1}^{K} \mathbb {E}[\tilde{n}_{k}(q_{0}+\tilde{x}_{k})-\log (1+e^{q_{0} + \tilde{x}_{k}})]\\
 & + \frac{-\tilde{K}}{2}\log (2\pi \sigma ^{2}_{\zeta })-\sum _{k\in \tilde{K}}\frac{\mathbb {E}\bigg [(\tilde{r}_{k}-\tilde{\gamma }_{0}-\tilde{\gamma }_{1}\tilde{x}_{k})^{2}\bigg ]}{2\sigma _{\zeta }^{2}}\\
 & + \frac{-K}{2}\log (2\pi \sigma ^{2}_{\psi })-\sum ^{K}_{k= 1}\frac{\mathbb {E}\bigg [(z_{k}-\tilde{\lambda }_{0}-\tilde{\lambda }_{1}\tilde{x}^{2}_{k})^{2}\bigg ]}{2\sigma _{\psi }^{2}}\\
 & + \frac{-K}{2}\log (2\pi \sigma ^{2}_{u})-\sum _{k=1}^{K}\frac{\mathbb {E}\bigg [(\tilde{x}_{k}-\tilde{x}_{k})^{2}\bigg ]}{2\sigma _{u}^{2}}.\tag{28}
\end{align*}

The algorithm iterates between the E-step and the M-step until the convergence.

## III. Results

**Fig. 2.**
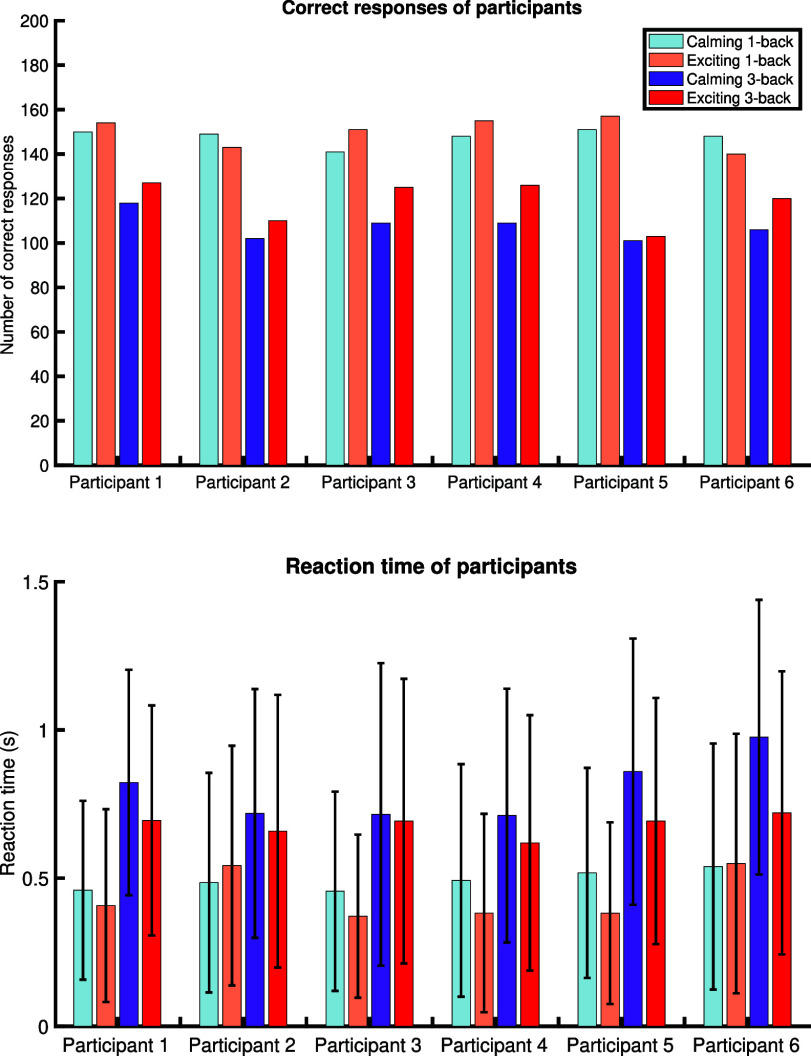
Number of correct responses and reaction time with respect to the type music and task for all participants. The top sub-panel presents the number of correct responses with respect to each session and n-back task block. The bottom sub-panel displays the average reaction times (the bars), and the error bars show the data within the first and third quartiles with respect to each session and n-back task block. The blue and red colors present the calming and exciting sessions, respectively. The darker colors with more intensity stand for the 3-back task blocks and the brighter ones present the 1-back task blocks.

The collected behavioral data at each session and type of n-back task can be found in Fig. 2. Mainly, the number of correct responses and the average reaction times for all participants can be found in Fig. 2. The blue bars correspond to a calming session, and the red bars correspond to an exciting session. The dark intense bars indicate the 3-back data, and the brighter ones stand for the 1-back task.

**Fig. 3.**
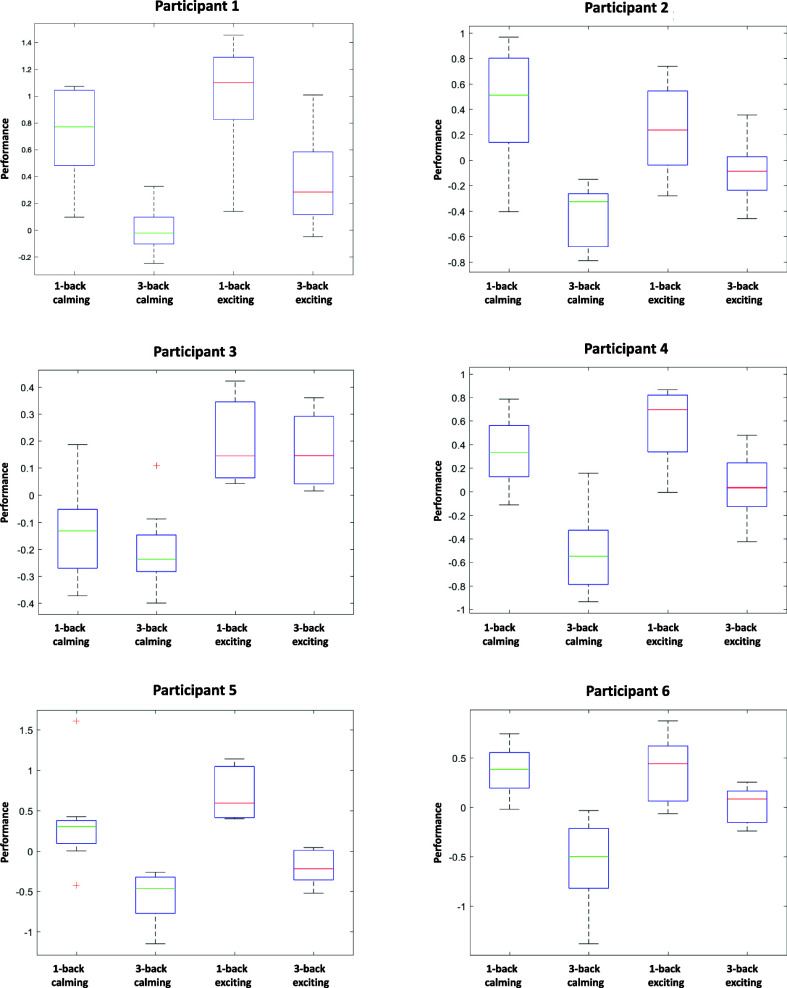
Distribution of performance state within different tasks and types of music. Each sub-figure shows the box plot of the performance state.

Fig. 3 presents the distribution of the performance state while different types of music were presented.

The estimated arousal state based on the MPP type observation for all participants are available in the supplementary information. Furthermore, we depict the distribution of average MPP-based arousal state within trials with respect to each tasks difficulty (supplementary information). We find the point-biserial correlation coefficients between the task difficulty and arousal by considering the task difficulty as dichotomous variable and the average arousal state within the trials as the continuous variable. In turn, the point-biserial correlation coefficients for participants 1 to 6 are 0.0603, -0.0268, 0.0166, 0.0150, -0.0206, and 0.0405, respectively.

**Fig. 4.**
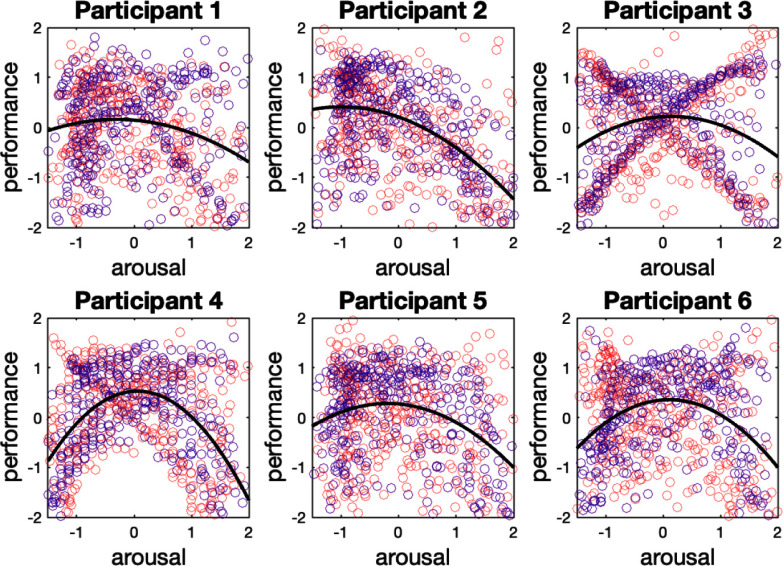
Arousal-performance diagram within the whole experiment. The x-axis represents the standard score of the estimated arousal derived from the marked point process estimator, and the y-axis stands for the standard score of the performance states. The red and blue data points show the observed pairs of arousal and performance withing the 3-back trials and 1-back trials, respectively. The black curve presents the fitted model.

In Fig. 4, we investigate the link between the estimated arousal from the MPP filter and the estimated performance. The blue and red points are associated with the 1-back and 3-back tasks, respectively. The data points can form an inverted-U shape for all participants with different coefficients. The $p$-values of the model parameters are provided in Table I. We can observe that the p-values for $\hat{\lambda }_{1}$ are statistically significant for all participants, while the coefficient $\hat{\lambda }_{2}$ is not statistically significant.

**Fig. 5.**
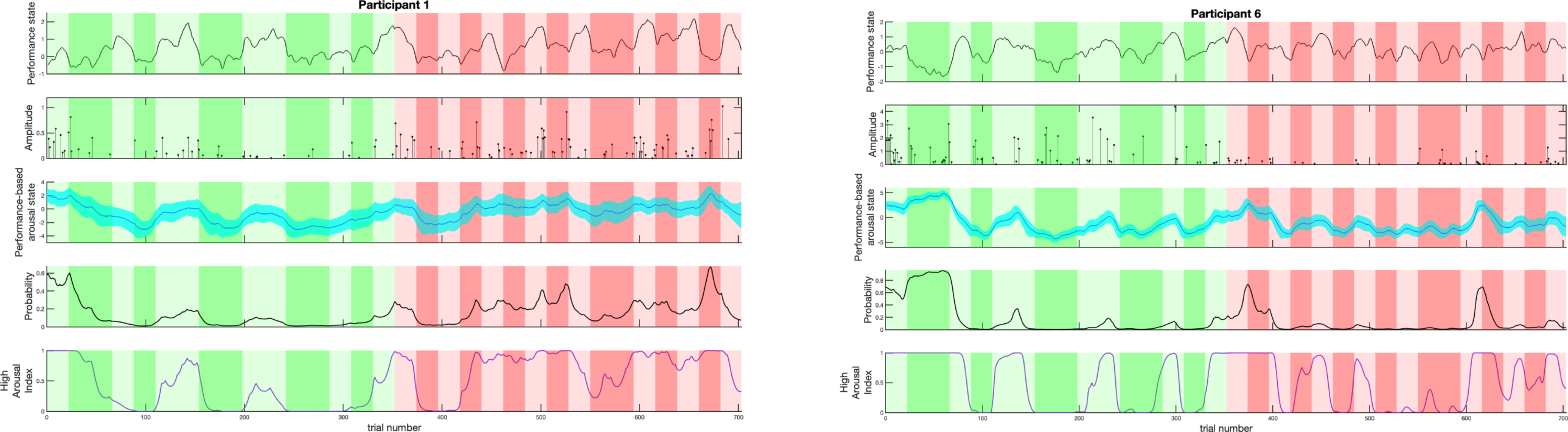
Arousal state estimation from performance-based decoder for two participants. The sub-panels of the figure at each column depict: The performance state signal ($z_{k}$); The average of the deconvolved neural impulses during trials –arousal events– ($\tilde{r}_{k}$); The estimated state ($\tilde{x}_{k}$) and its 95% confidence limits; The probability of impulse occurrence ($\phi _{k}$); The high arousal index. The background colors in each sub-panel depict: The 1-back task during the calming session (light green); The 3-back task during the calming session (dark green); The 1-back task during the exciting session (light red); The 3-back task during the exciting session (dark red).

We represent the performance-based arousal estimator results for participants 1 and 6 in Fig. 5. The first two subplots at each column present the observed performance and the average of arousal events at each trial, which together comprise the decoder observation. The third and fourth subplots show the estimated arousal state and the probability of observing arousal events, respectively. To study the personalized trajectories of arousal, we formulate a high arousal index (HAI) to generalize the estimated arousal level of participants ($\tilde{x}_{k}$) [14]. The HAI can be calculated from $p(\tilde{x}_{k}>x_{\text{threshold}})$, where the threshold has been set to the median of the state values. It indicates the probability that a binary event occurs more than just by chance over the experiment. The performance-based arousal state estimation results for other participants can be found in the supplementary materials.

**Fig. 6.**
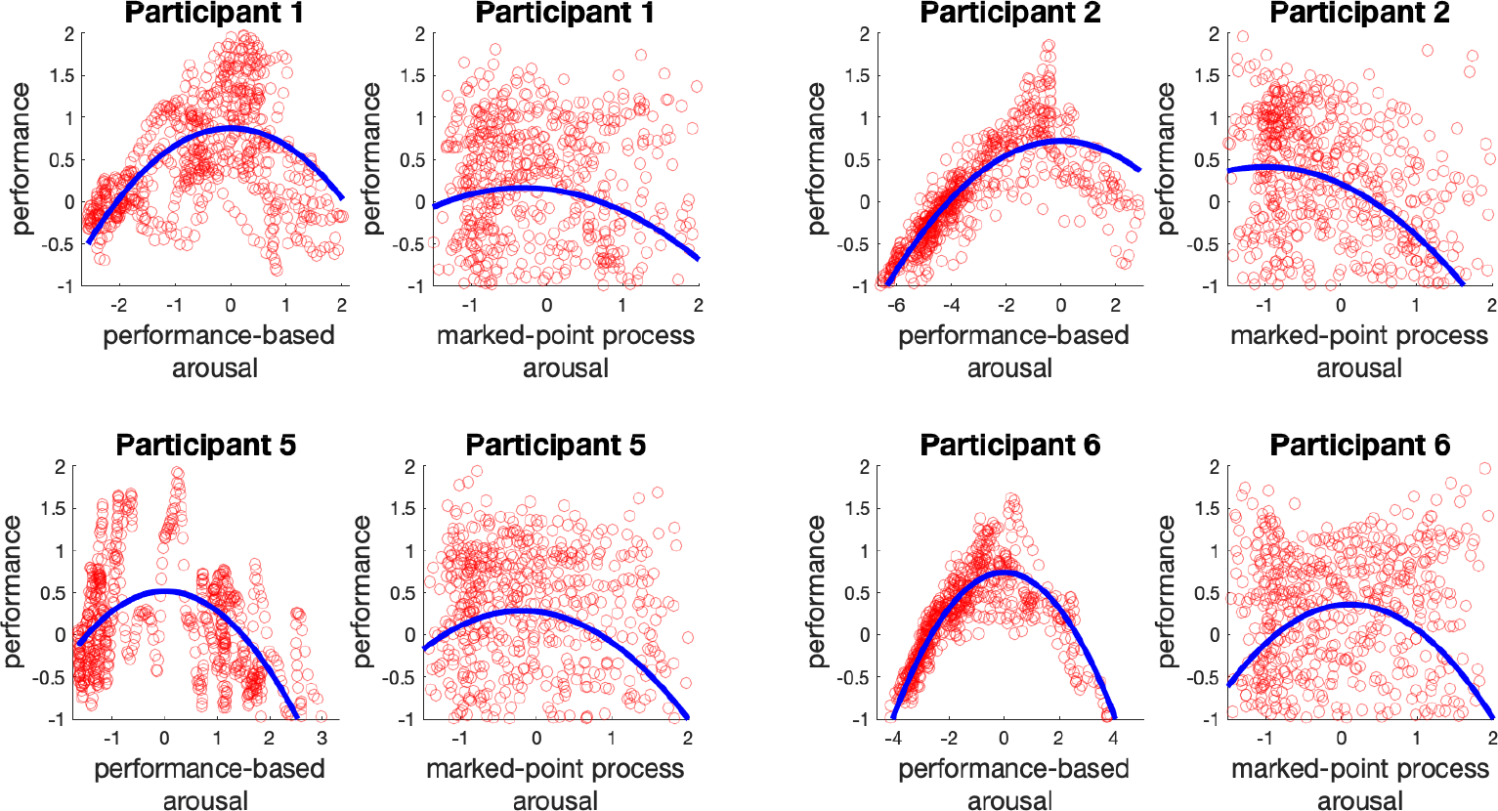
Arousal-performance diagram comparison. In each pair of arousal-performance diagrams, the left sub-panel displays the arousal-performance data points constructed from the performance-based arousal estimator; The right subplot demonstrates the standard score of data points derived from the marked point process arousal estimator and performance state decoder. The blue curves show the fitted model for the corresponding data points (red).

In Fig. 6, we compare the arousal-performance link derived from the performance-based arousal state and the estimated arousal from the MPP decoder.

**Fig. 7.**
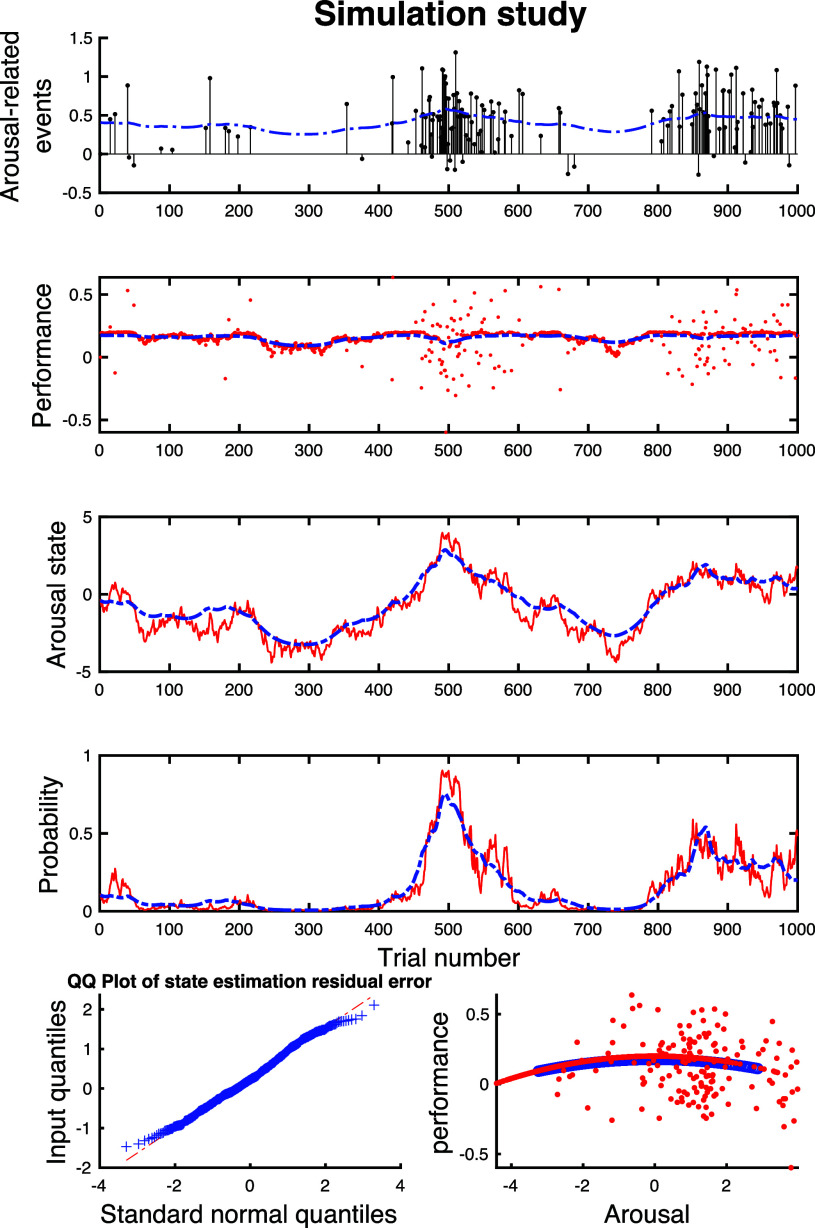
Performance-based arousal state estimation on simulated data. The sub-panels of the figure depict, in turns: Simulated arousal events with their amplitudes (black impulses) and reconstructed $\tilde{r}_{k}$ (blue); Simulated performance state (red dots) and reconstructed one (blue); Simulated arousal state as ground truth (red) and estimated state (blue); Simulated probability of observing an arousal event (red) and estimated one (blue); The quantile-quantile (QQ) plot of arousal state residual error; The arousal-performance link such that the simulated data points are shown by red dots and the estimated ones are presented by blue dots.

According to the proposed models, we simulate sets of internal states (Fig. 7). The subplots of Fig. 7 present simulated arousal events with their amplitudes and reconstructed $\tilde{r}_{k}$, simulated performance state and reconstructed one, simulated arousal state (ground truth) and estimated one, simulated probability of observing an arousal event and estimated one, the quantile-quantile (QQ) plot of arousal state residual error, and the arousal-performance link, in turns. The R-squared value for the estimated arousal state is 0.8541.

## IV. Discussion

In most of the affective studies, the absence of ground truth resulted in implicit evaluation and validation of estimated arousal. An instance of such implicit evaluation would be using experiment information such as presented emotional stimuli to evaluate the decoder's outcome [33]. In this study, the experiment was designed with two types of task blocks (i.e., 1-back and 3-back tasks) and two different background music. We evaluate and discuss the decoded arousal and performance state with respect to each session and each type of task. Particularly, since the high cognitive load can reduce cognitive performance, we would expect to see lower performance levels in 1-back trials compared to 3-back trials in the decoded performance [34]. Furthermore, due to the exciting content of the selected music in the second session, the participant may experience a higher level of arousal within the second session compared to the first session, and the decoded arousal state may be evaluated accordingly [25], [35], [36].

The collected behavioral data that is used as an observation to decode the performance is presented in Fig. 2; the decoded performance is demonstrated in Fig. 3. Apart from Participant 2, the higher number of correct responses, as well as the faster reaction time, can be seen within the second session. These higher performance metrics in the second session are aligned with the decoded performance state. Also, the low performance during the 3-back and high performance during the 1-back task blocks are noticeable in both the decoded performance and the recorded behavioral data. Overall, there is a decent agreement between the estimated performance state and the observed performance metrics. Considering the variation of performance metrics within the sessions, we may notice that the performance variation from calming to exciting in 3-back task blocks is higher than the 1-back ones. One possible explanation for the observed behavior is that participants already perform well enough within the 1-back tasks to the point that no considerable difference can be made by changing the condition [37]. This is aligned with the findings in [38], where participants performed near ceiling level at 1-back regardless of the applied training.

In general, our findings present higher performance matrices associated with the exciting session for five out of six participants. One may argue that the improved performance is an indication of the arousal establishment within the desired range using music. However, other factors such as, the learning effect, the nature of the task, and the participant's baseline can be involved which hinder us from drawing any definite conclusion. Particularly, it should be noted that the exciting session was implemented as the second session; it is possible that the participant outperformed in the second session due to learning the task [39]. Hence, while it might be viable to impact the performance via personalized music, further studies are needed to make any solid judgment on the impact of music on performance. Specifically, including a control group in this context can provide a better insight into the impact of music as well as the presence of learning.

It is worth highlighting participant 2 as the only participant that presents a lower performance within the second session. This may indicate that the exciting component of music does not improve this person's performance. Perhaps the exciting music makes the person excessively aroused while the person's ideal arousal level is located within the lower arousal range. Another interpretation from the observed trend would be the absence of plausible learning. Looking into the arousal-performance link for participant 2, Table I and Fig. 4 reveal a relatively strong linear component in the arousal-performance relationship. Commonly, the linear arousal-performance link can be expected when a person does not experience high enough arousal [40]. However, for participant 2, we have a different scenario in which the presence of high performance in the low arousal range and the absence of high performance in the high arousal range region is seen. A linear model can describe the arousal-performance link in such cases where either the rise or decay of the performance is presented solely. Nevertheless, if the rise or decay occurs exponentially, the exponential model also can be a good fit.

In this research, to avoid the potential reduced statistical power [41], we consider all the data collected during the experiment to perform the regression analysis and identify the presence of Yerkes-Dodson law. One may interpret the presence of Yerkes-Dodson law from the presented analysis. One crucial point that needs to be addressed is the extent to which the task difficulty and distribution impact the underlying arousal and, subsequently, the observed arousal-performance link. The point-biserial correlation coefficients do not reveal a significant association between the n-back task difficulty and the arousal, which agrees with the findings in [42]. However, we should keep in mind that in this research, the arousal state is derived from the skin conductance signal, and employing other physiological signals as an arousal index may produce a different outcome, in which task difficulty plays a confounding role [42], [43].

The participants were asked to provide calming and exciting music with no quantification or rating of the elicited emotion. In particular, the applied music was meant to have a person-specific impact rather than equal emotion elicitation across the whole sample size. Hence, we should be cautious in generalizing the findings. Instead, we may investigate the results with an individualized viewpoint. Thus, we use the participant's baseline to present person-specific metrics of arousal and performance (i.e., HAI and HPI), which can be found in the supplementary information.

While the applied personalized music mimics the personalized closed-loop architecture, it can induce the impact of familiarity on arousal and performance. One possible way to reduce the effect of music familiarity and preserve the personalized nature of the music intervention is to employ new generative deep-learning models and produce new music based on the participant's preferences person [44].

Using the performance-based arousal decoder, we can obtain the arousal level corresponding to the performance of each trial (Fig. 5). The performance-based arousal decoder can benefit from having a performance as one of the observations. Specifically, for participant 6, in spite of the few observed ANS activations during the exciting session, the higher baseline of performance in the exciting session would prevent an excessive drop of arousal. As it can be seen in Fig. 6, the arousal-performance links derived from the performance-based model (Fig. 5) tend to maintain the inverted quadratic shape and follow the inverted-U law. Additionally, since we are using both behavioral data and skin conductance signal for the performance-based arousal decoder, the results would be less affected by the possible artifacts from the skin conductance signal recording, solely.

The simulation study illustrates a decent performance of the proposed decoder. Specifically, the R-squared value and QQ plot display an agreement between the decoded state trajectory and the ground truth. While the Bayesian state-space approach is a powerful estimation tool, there are cases that might suffer from the overfitting issue [33]. This simulation study (first two subplots of Fig. 7) demonstrates that the estimates do not overfit to either the provided MPP observation or the continuous observation.

## V. Conclusion

In the studied n-back experiment, two types of music were presented to investigate the potential of developing a safe neurofeedback via music. Using a state-space modeling approach, we decode cognitive arousal and performance states of participants. To obtain a better insight into the arousal and performance relationship, we evaluate the plausible Yerkes—Dodson law via regression analysis. The existence of Yerkes—Dodson law would be one possible interpretation from the observed results while the small sample size and a lack of a control group would hinder us from establishing a definitive conclusion.

Our study would shed light on the primary idea of enhancing cognitive performance and shifting one's arousal using music. It might be feasible to impact the arousal and performance via music [45]. However, it should be highlighted that several factors, such as the learning effect, the nature of the task, the participant's baseline, and the type of applied music, can impact the outcome. Hence, a more comprehensive experiment with a larger sample size, control group, shuffled cognitive tasks, and various types of music would be helpful for having a settled resolution on the music's effect on arousal and performance.

We design a performance-based arousal decoder that estimates the arousal level of individuals based on their performance. This type of decoder conforms to the Yerkes—Dodson law. The ultimate goal of this performance-based arousal decoder is to be implemented within safe closed-loop systems, and the proposed decoder can be further investigated in different behavioral experiments [46], [47], [48]. In the future, we aim to test decoders in different experiments and quantify the arousal and performance in various environments. Also, given the developed performance-based arousal decoder, informative signals such as pupil size can be used in parallel with the skin conductance signal to decode the hidden arousal and evaluate the performance of the decoder [49].

*Supplementary Materials:* Additional figures, details of the methods, and further discussion are provided in supplementary materials.

Supplementary materials

*Conflict of Interest Statement:* RTF and MRA are co-inventors of a patent application filed by the University of Houston reltaed to this research.

*Author Contributions:* Conceptualization: RTF; Formal analysis: SK; Funding acquisition: RTF; Investigation: SK, MRA, MT, and RTF; Methodology: SK, MRA, and RTF; Validation: SK; Visualization: SK, MRA, and MT; Writing — original draft: SK; Writing — review and editing: SK, MRA, MT, and RTF.
