# Supplementary material for: Bayesian Inference of Hidden Cognitive Performance and Arousal States in Presence of Music
Source: IEEE Open J Eng Med Biol. 2024 Mar 18;5:627–36. doi: 10.1109/OJEMB.2024.3377923 (PMC11342937; doi:10.1109/OJEMB.2024.3377923)
Supplement: Supplementary materials [file supp1-3377923.pdf]

Supplementary Materials

Bayesian Inference of Hidden Cognitive Performance and Arousal States in Presence of Music

Saman Khazaei, *Student Member, IEEE*, Md Rafiul Amin, *Member, IEEE*,Maryam Tahir, and Rose T. Faghih, *Senior Member, IEEE*

I. Inference of Brain Activation from Skin Conductance Measurements

The measured skin conductance during the experiment is evaluated as a summation of two components: (1) a tonic component and (2) a phasic component, which fluctuates faster compared to the tonic part [1]–[3]. The tonic component is associated with the body's thermoregulation and general arousal. The phasic component can be specified as the convolution between the neural impulse train due to ANS activation and the physiological system response [2], [3]. To recover the neural impulse train from the raw skin conductance signal, we perform a deconvolution. First, we pre-process the data similar to [4], [5]. Second, we use a low-pass filter at 0.5 Hz for filtering our data. Then, we remove the tonic component using cvxEDA [6]. Employing a coordinate descent approach, we deconvolve the extracted phasic component to estimate the sparse arousal events due to ANS activation [4], [7]–[9].

II. A Marked Point Process State-Space Decoder for Arousal

Simultaneously, we estimate the unknown parameters $\theta_A = \{\sigma_\varepsilon^2, \hat{\gamma}_0, \hat{\gamma}_1, \sigma_v^2\}$, and hidden state $\hat{x}_j$, within the expectation-maximization framework.

A. E-Step

The expectation step is composed of two subsections. Forward filtering and backward smoothing, respectively. Given the observations $R^J = \{(n_1, r_1), ..., (n_J, r_J)\}$ up to time J, E-step equations can be derived. Forward filtering formulation can be obtained from:

Predict:

$$\hat{x}_{j|j-1} = \hat{x}_{j-1|j-1}, \tag{1}$$

$$\hat{\sigma}^2_{j|j-1} = \hat{\sigma}^2_{j-1|j-1} + \sigma^2_\varepsilon, \tag{2}$$

Update:

if $n_j = 0$

$$\hat{x}_{j|j} = \hat{x}_{j|j-1} + \hat{\sigma}^2_{j|j-1}(n_j - a_{j|j}), \tag{3}$$

$$\hat{\sigma}^2_{j|j} = \left[\frac{1}{\hat{\sigma}^2_{j|j-1}} + a_{j|j}(1 - a_{j|j})\right]^{-1}, \tag{4}$$

if $n_j = 1$

$$C_j = \frac{\hat{\sigma}^2_{j|j-1}}{\hat{\gamma}_1^2 \hat{\sigma}^2_{j|j-1} + \sigma_v^2}, \tag{5}$$

$$\hat{x}_{j|j} = \hat{x}_{j|j-1} + C_j \Big[\sigma_v^2 (n_j - a_{j|j}) + \hat{\gamma}_1 (r_j - \hat{\gamma}_0 - \hat{\gamma}_1 \hat{x}_{j|j-1})\Big], \tag{6}$$

$$\hat{\sigma}^2_{j|j} = \left[\frac{1}{\sigma^2_{j|j-1}} + a_{j|j}(1 - a_{j|j}) + \frac{\gamma_1^2}{\sigma_v^2}\right]^{-1}. \tag{7}$$

Since $\hat{x}_{j|j}$ appears on both sides of (3) and (6), we can solve for $\hat{x}_{j|j}$ by applying numerical methods such as Newton-Raphson.

In order to implement the backward smoothing, we reverse the direction and improve the estimation by calculating a set of smoothed mean and variance:

$$\hat{A}_j = \frac{\hat{\sigma}^2_{j|j}}{\hat{\sigma}^2_{j+1|j}}, \tag{8}$$

$$\hat{x}_{j|j} = \hat{x}_{j|j} + \hat{A}_j (\hat{x}_{j+1|J} - \hat{x}_{j+1|j}), \tag{9}$$

$$\hat{\sigma}^2_{j|j} = \hat{\sigma}^2_{j|j} + \hat{A}_j^2 (\hat{\sigma}^2_{j+1|j} - \hat{\sigma}^2_{j+1|j}). \tag{10}$$

Similar to [10] and [11], we derive the expected values of $\hat{x}_j^2$, and $\hat{x}_j \hat{x}_{j-1}$ using the following,

$$\mathbb{E}[\hat{x}_j^2] = \hat{x}^2_{j|j} + \hat{\sigma}^2_{j|j}, \tag{11}$$

$$\mathbb{E}[\hat{x}_{j+1}\hat{x}_j] = x_{j+1|J}\hat{x}_{j|j} + \hat{A}_j \hat{\sigma}^2_{j+1|j}. \tag{12}$$

B. M-Step

At the M-step, we consider $\hat{J} = \{j | n_j = 1\}$ to specify the locations of neural impulses. Using the E-step results, we obtain the log-likelihood function Q_1 and we find the unknown parameters such that they maximize it. The Q_1 function would be

$$Q_1 = \sum_{j=1}^{J} \mathbb{E}[n_j(\beta + \hat{x}_j) - \log(1 + e^{\beta + \hat{x}_j})] + \frac{-\hat{J}}{2}\log(2\pi\sigma_v^2) - \sum_{j \in \hat{J}} \frac{\mathbb{E}\left[(r_j - \hat{\gamma}_0 - \hat{\gamma}_1 \hat{x}_j)^2\right]}{2\sigma_v^2} + \frac{-J}{2}\log(2\pi\sigma_\varepsilon^2) - \sum_{j=1}^{J} \frac{\mathbb{E}\left[(\hat{x}_j - \hat{x}_{j-1})^2\right]}{2\sigma_\varepsilon^2}. \tag{13}$$

III. A State-Space Decoder for Performance

The unknown parameters vector of the performance state model $\theta_P = \{\rho, \sigma_w^2, \alpha_0, \alpha_1, \sigma_\delta^2\}$, and the performance state z_k can be estimated through EM approach.

A. E-Step

The E-step equations are slightly different from the arousal state decoder. The forward filter equations are

Predict:

$$z_{k|k-1} = \rho z_{k-1|k-1} \tag{14}$$
$$s^2_{k|k-1} = \rho^2 s^2_{k-1|k-1} + \sigma_w^2$$

Update:

$$z_{k|k} = z_{k|k-1} + \frac{s^2_{k|k-1}}{\alpha_1^2 s^2_{k|k-1} + \sigma_\delta^2}\Big[\sigma_\delta^2 (m_k - p_{k|k}) \tag{15}$$
$$+ \alpha_1 (l_k - \alpha_0 - \alpha_1 z_{k|k-1})\Big]$$
$$s^2_{k|k} = \left[\frac{1}{s^2_{k|k-1}} + p_{k|k}(1-p_{k|k}) + \frac{\alpha_1^2}{\sigma_\delta^2}\right]^{-1} \tag{16}$$

By reversing the direction, we construct the smoother.

$$B_k = \rho \frac{s^2_{k|k}}{s^2_{k+1|k}}, \tag{17}$$
$$z_{k|K} = z_{k|k} + B_k (z_{k+1|K} - z_{k+1|k}), \tag{18}$$
$$s^2_{k|K} = s^2_{k|k} + B_k^2 (s^2_{k+1|K} - s^2_{k+1|k}). \tag{19}$$

The expected values of z_k^2, and $z_k z_{k-1}$ are ,

$$\mathbb{E}[z_k^2] = z^2_{k|K} + s^2_{k|K}, \tag{20}$$
$$\mathbb{E}[z_{k+1} z_k] = z_{k+1|K} z_{k|K} + B_k s^2_{k+1|K}. \tag{21}$$

B. M-Step

For the M-step formulation, the expected log-likelihood function would be,

$$Q_2 = \sum_{k=1}^{K} \mathbb{E}[m_k(\mu + z_k) - \log(1 + e^{\mu + z_k})] \tag{22}$$
$$+ \frac{-K}{2}\log(2\pi\sigma_\delta^2) - \sum_{k=1}^{K} \frac{\mathbb{E}\left[(l_k - \alpha_0 - \alpha_1 z_k)^2\right]}{2\sigma_\delta^2}$$
$$+ \frac{-K}{2}\log(2\pi\sigma_w^2) - \sum_{k=1}^{K} \frac{\mathbb{E}\left[(z_k - z_{k-1})^2\right]}{2\sigma_w^2}.$$

IV. Dataset

Prior to the experiment, the participants were provided with a consent form to participate in a human research study, and the experimenter requested them to provide personalized music. Therefore, they were familiar with the music while nuanced in the n-back task. The music requested by the experimenter was annotated as "calming" and "vexing" music, while the content of the provided music by participants was calming and exciting. Hence, in this research, we name the sessions based on the content of the music.

V. Results

The left columns of Fig. 1 to Fig. 6 display the outcome of the marked point process (MPP) arousal decoder for each participant. As described in the second sub-panels, neural impulses due to ANS activations can be captured by employing the deconvolution method. There is a direct relationship between the estimated probability and estimated state such that when the arousal state ($\hat{x}_j$) increases, the probability of spike occurrence would increase as well.

The right columns of Fig. 1 to Fig. 6 show the performance state estimation results for each participant. Given the continuous reaction time τ_k (black dots in the first sub-plot) and, the corresponding binary observations m_k (second sub-plot), we quantify the performance state. The reconstructed reaction time (black curve in first sub-plot) has been calculated by plugging the performance states and model parameters into $\tilde{t}_k = e^{(\alpha_0 + \alpha_1 z_k)}$. We formulate the high performance index (HPI) to generalize the performance and express the probability of deliberate correct responses such that it can be derived from $p(z_k > z_{\text{threshold}})$ where the threshold has been set to the median of the state values. Also, we present the distribution of average arousal state within trials with respect to each task difficulty in Fig. 7 where boxplots are available.

We study how the HPI changes from one task to another task according to the background music. The averages of the HPI transitions have been shown in Fig. 8. Note that the experiment does not include the 1-back to 1-back transition during the exciting section.

Figure 9 demonstrates the performance-based arousal decoder's outcome for participants 2 and 5. Given the observation vector that includes the arousal events and estimated performance state (first two subplots of Fig. 9), we decode the hidden arousal (third subplot of Fig. 9). Participants 3 and 4 are excluded from the performance-based arousal state estimation due to the unusual smoothness of skin conductance response.

VI. Discussion

One possible approach for setting the initial values in the EM algorithm is to consider the log-likelihood function and run the algorithm multiple times with random initial conditions and eventually, find a set of initial conditions that produces the highest log-likelihood function as the optimal set. This approach may have a high computational cost since we have 20 model parameters. Hence, we employ the previous investigations and select the initial values based on the previous algorithm execution.

Given the skin conductance signal and the recovered ANS activation, we observe that participants 3 and 4 skin conductance responses are highly smooth such that the recovered arousal events are extremely sparse. Hence, we exclude

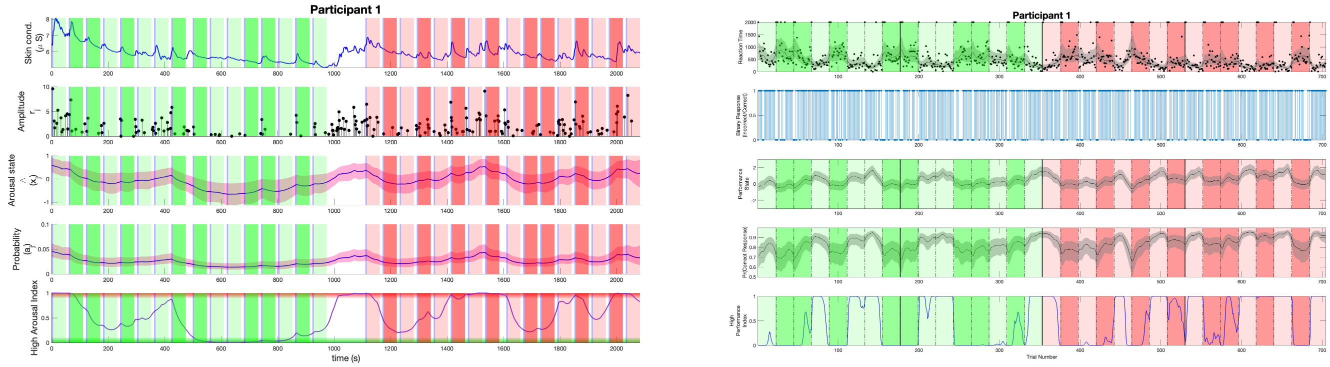

Fig. 1. **Arousal and performance states estimation results for one participant.** The left column represents the arousal state estimation outcome such that the sub-panels respectively depict: the skin conductance signal; the deconvolved neural impulses from ANS activation; the estimated state and its 95% confidence limits; the probability of the impulse occurrence and its 95% confidence limits; high arousal index or HAI. The right column represents the performance state estimation outcome such that the sub-panels respectively depict: the reaction time (black dots), reconstructed reaction time (black curve) and its 95 % confidence limits; correct/incorrect response; the state estimation and its 95 % confidence limits; the probability of correct response and its 95% confidence limits; high performance index or HPI. The background colors in each sub-panel mark: the 1-back task during the calming session (light green); the 3-back task during the calming session (dark green); the 1-back task during the exciting session (light red); the 3-back task during the exciting session (dark red).

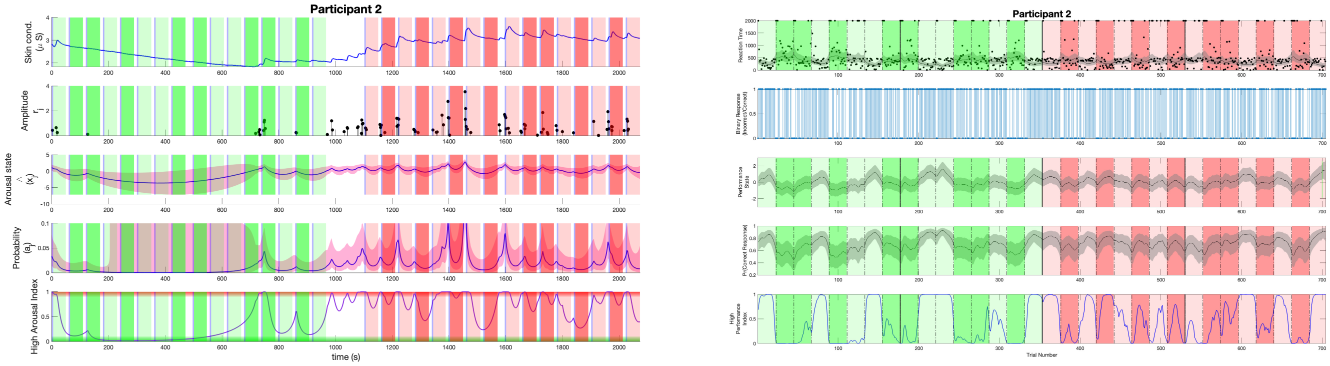

Fig. 2. **Arousal and performance states estimation results for one participant.** The left column represents the arousal state estimation outcome such that the sub-panels respectively depict: the skin conductance signal; the deconvolved neural impulses from ANS activation; the estimated state and its 95% confidence limits; the probability of the impulse occurrence and its 95% confidence limits; high arousal index or HAI. The right column represents the performance state estimation outcome such that the sub-panels respectively depict: the reaction time (black dots), reconstructed reaction time (black curve) and its 95 % confidence limits; correct/incorrect response; the state estimation and its 95 % confidence limits; the probability of correct response and its 95% confidence limits; high performance index or HPI. The background colors in each sub-panel mark: the 1-back task during the calming session (light green); the 3-back task during the calming session (dark green); the 1-back task during the exciting session (light red); the 3-back task during the exciting session (dark red).

participants 3 and 4 from evaluating the performance-based arousal decoder. By visual inspection, we can see that HAI and state values are higher at the exciting region for the first three participants; the baseline of the HPI and state values are higher in the red region for participants (except participant 4). This emphasizes the music's impact on both arousal and performance levels, which is in agreement with the findings in [12].

According to Fig. 8, for all participants, the elevation in performance can be detected when a participant switches from the 3-back to the 1-back task. Conversely, switching between the 1-back to the 3-back task results in the HPI drop. This trend is consistent with our previous expectation which states that the 1-back task is easier to perform than the 3-back task. According to the performance state value distribution of the participant 3, the performance state values are negative at the calming session and suddenly, we have a sharp increase in the exciting session; Since the $z_{threshold}$ for calculating HPI is defined as the median of the states during both sessions, this can give rise to have a very low and steady HPI level for the calming session, and extremely steady and high value for the exciting session. From the mathematical perspective, given the threshold, the probability of having the state higher than z_{th} at the calming session is extremely low while it would be extremely high at the exciting session.

In the simulation study, we consider 8 parameters to simulate 1000 data points for each variable. The parameters are specified based on the experimental results. One of the crucial simulated

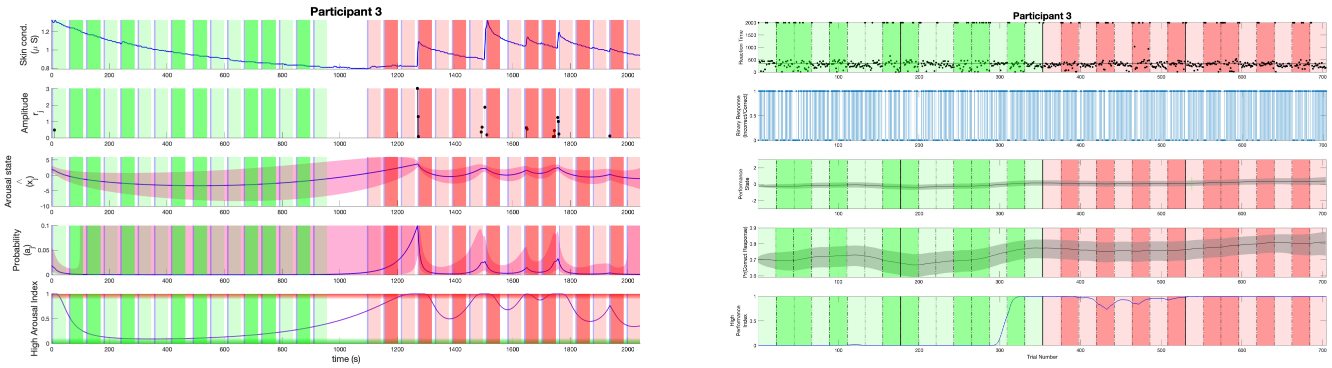

Fig. 3. **Arousal and performance states estimation results for one participant.**The left column represents the arousal state estimation outcome such that the sub-panels respectively depict: the skin conductance signal; the deconvolved neural impulses from ANS activation; the estimated state and its 95% confidence limits; the probability of the impulse occurrence and its 95% confidence limits; high arousal index or HAI. The right column represents the performance state estimation outcome such that the sub-panels respectively depict: the reaction time (black dots), reconstructed reaction time (black curve) and its 95 % confidence limits; correct/incorrect response; the state estimation and its 95 % confidence limits; the probability of correct response and its 95% confidence limits; high performance index or HPI. The background colors in each sub-panel mark: the 1-back task during the calming session (light green); the 3-back task during the calming session (dark green); the 1-back task during the exciting session (light red); the 3-back task during the exciting session (dark red).

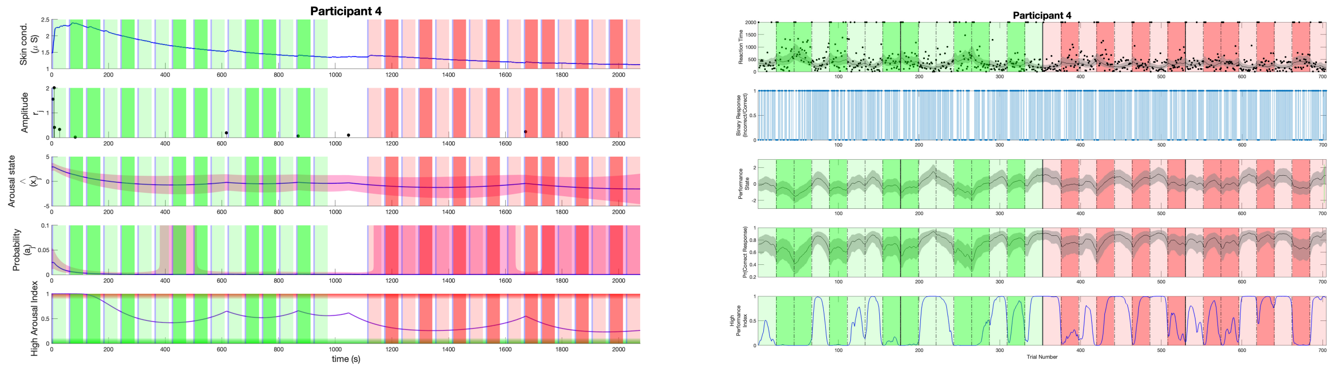

Fig. 4. **Arousal and performance states estimation results for one participant.** The left column represents the arousal state estimation outcome such that the sub-panels respectively depict: the skin conductance signal; the deconvolved neural impulses from ANS activation; the estimated state and its 95% confidence limits; the probability of the impulse occurrence and its 95% confidence limits; high arousal index or HAI. The right column represents the performance state estimation outcome such that the sub-panels respectively depict: the reaction time (black dots), reconstructed reaction time (black curve) and its 95 % confidence limits; correct/incorrect response; the state estimation and its 95 % confidence limits; the probability of correct response and its 95% confidence limits; high performance index or HPI. The background colors in each sub-panel mark: the 1-back task during the calming session (light green); the 3-back task during the calming session (dark green); the 1-back task during the exciting session (light red); the 3-back task during the exciting session (dark red).

variables is a marked-point process random variable in this paradigm. While we consider the arousal events to be associated with a positive amplitude in modeling the neural firing process, it is unavoidable to have negative marked values in simulating data while positive impulses would be informative enough to perform a descent state estimation. There is a good agreement between the estimated arousal and the ground truth. In terms of the arousal-performance link, the estimated data points follow a similar trend as the ground truth while the simulated arousal-performance link carries an outlier.

In order to gain a better understanding of the internal brain states (arousal and performance states) in different conditions, we can design a more advanced experiment by utilizing unfamiliar background music generated from deep neural networks, shuffling them, and considering the combination of tasks similar to [13] to avoid the habitual behavior. Furthermore, we plan to consider different noise models for linking cognitive arousal to cognitive performance to better capture the inverted-U relationship between these two brain states.

References

[1] M. R. Amin, D. S. Wickramasuriya, and R. T. Faghih, "A wearable exam stress dataset for predicting grades using physiological signals," in *2022 IEEE Healthcare Innovations and Point of Care Technologies (HI-POCT)*, pp. 30–36, IEEE, 2022.

[2] M. R. Amin and R. T. Faghih, "Identification of sympathetic nervous system activation from skin conductance: A sparse decomposition approach with physiological priors," *IEEE Transactions on Biomedical Engineering*, vol. 68, no. 5, pp. 1726–1736, 2020.

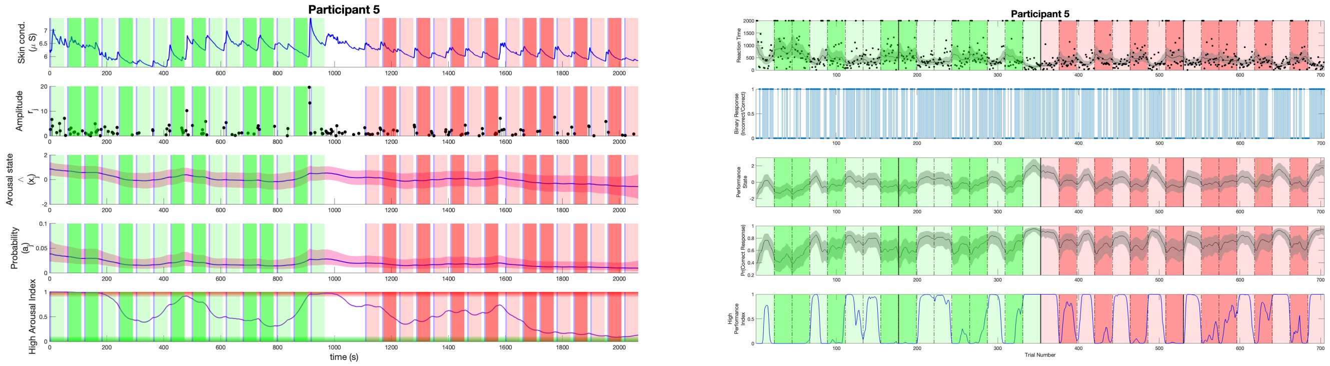

Fig. 5. **Arousal and performance states estimation results for one participant.** The left column represents the arousal state estimation outcome such that the sub-panels respectively depict: the skin conductance signal; the deconvolved neural impulses from ANS activation; the estimated state and its 95% confidence limits; the probability of the impulse occurrence and its 95% confidence limits; high arousal index or HAI. The right column represents the performance state estimation outcome such that the sub-panels respectively depict: the reaction time (black dots), reconstructed reaction time (black curve) and its 95 % confidence limits; correct/incorrect response; the state estimation and its 95 % confidence limits; the probability of correct response and its 95% confidence limits; high performance index or HPI. The background colors in each sub-panel mark: the 1-back task during the calming session (light green); the 3-back task during the calming session (dark green); the 1-back task during the exciting session (light red); the 3-back task during the exciting session (dark red).

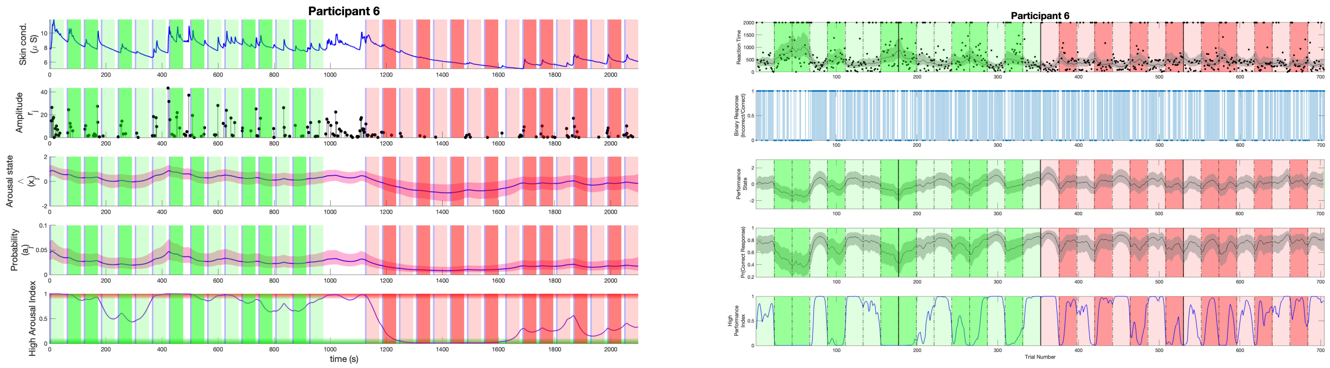

Fig. 6. **Arousal and performance states estimation results for one participant.** The left column represents the arousal state estimation outcome such that the sub-panels respectively depict: the skin conductance signal; the deconvolved neural impulses from ANS activation; the estimated state and its 95% confidence limits; the probability of the impulse occurrence and its 95% confidence limits; high arousal index or HAI. The right column represents the performance state estimation outcome such that the sub-panels respectively depict: the reaction time (black dots), reconstructed reaction time (black curve) and its 95 % confidence limits; correct/incorrect response; the state estimation and its 95 % confidence limits; the probability of correct response and its 95% confidence limits; high performance index or HPI. The background colors in each sub-panel mark: the 1-back task during the calming session (light green); the 3-back task during the calming session (dark green); the 1-back task during the exciting session (light red); the 3-back task during the exciting session (dark red).

[3] M. R. Amin and R. T. Faghih, "Tonic and phasic decomposition of skin conductance data: A generalized-cross-validation-based block coordinate descent approach," in *2019 41st Annual International Conference of the IEEE Engineering in Medicine and Biology Society (EMBC)*, pp. 745–749, IEEE, 2019.

[4] D. S. Wickramasuriya, M. Amin, R. T. Faghih, *et al.*, "Skin conductance as a viable alternative for closing the deep brain stimulation loop in neuropsychiatric disorders," *Frontiers in neuroscience*, vol. 13, p. 780, 2019.

[5] M. R. Amin and R. T. Faghih, "Sparse deconvolution of electrodermal activity via continuous-time system identification," *IEEE Transactions on Biomedical Engineering*, vol. 66, no. 9, pp. 2585–2595, 2019.

[6] A. Greco, G. Valenza, A. Lanata, E. P. Scilingo, and L. Citi, "cvxeda: A convex optimization approach to electrodermal activity processing," *IEEE Transactions on Biomedical Engineering*, vol. 63, no. 4, pp. 797–804, 2015.

[7] J. X. Genty, M. R. Amin, N. D. Shaw, E. Klerman, and R. T. Faghih, "Sparse deconvolution of pulsatile growth hormone secretion in adolescents," *IEEE/ACM Transactions on Computational Biology and Bioinformatics*, 2021.

[8] R. T. Faghih, "From physiological signals to pulsatile dynamics: A sparse system identification approach," in *Dynamic Neuroscience*, pp. 239–265, Springer, 2018.

[9] R. T. Faghih, M. A. Dahleh, G. K. Adler, E. B. Klerman, and E. N. Brown, "Quantifying pituitary-adrenal dynamics and deconvolution of concurrent cortisol and adrenocorticotropic hormone data by compressed sensing," *IEEE Transactions on Biomedical Engineering*, vol. 62, no. 10, pp. 2379–2388, 2015.

[10] D. S. Wickramasuriya and R. T. Faghih, "A marked point process filtering approach for tracking sympathetic arousal from skin conductance," *IEEE Access*, vol. 8, pp. 68499–68513, 2020.

[11] A. C. Smith, L. M. Frank, S. Wirth, M. Yanike, D. Hu, Y. Kubota, A. M. Graybiel, W. A. Suzuki, and E. N. Brown, "Dynamic analysis of learning in behavioral experiments," *J. Neuroscience*, vol. 24, no. 2, pp. 447–461, 2004.

[12] J. A. Lehmann and T. Seufert, "The influence of background music on learning in the light of different theoretical perspectives and the role of working memory capacity," *Frontiers in psychology*, vol. 8, p. 1902,

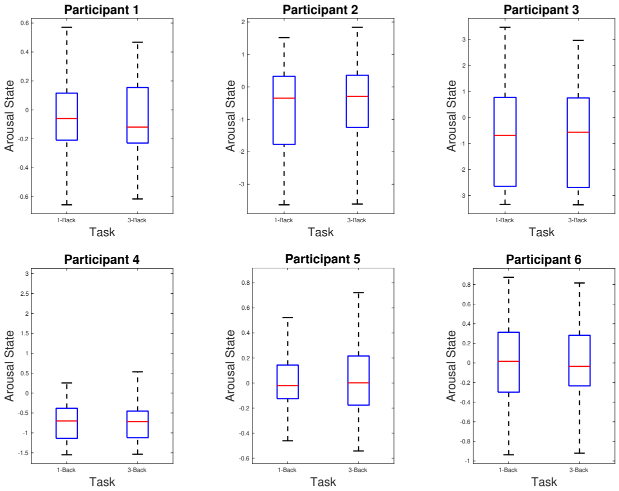

Fig. 7. **Distribution of average arousal state within trials with respect to tasks.** Each sub-figure shows the box plot of the average arousal state data within the trials with respect to 1-back and 3-back task blocks.

2017.

[13] A. Unni, K. Ihme, M. Jipp, and J. W. Rieger, "Assessing the driver's current level of working memory load with high density functional near-infrared spectroscopy: a realistic driving simulator study," *Frontiers in human neuroscience*, vol. 11, p. 167, 2017.

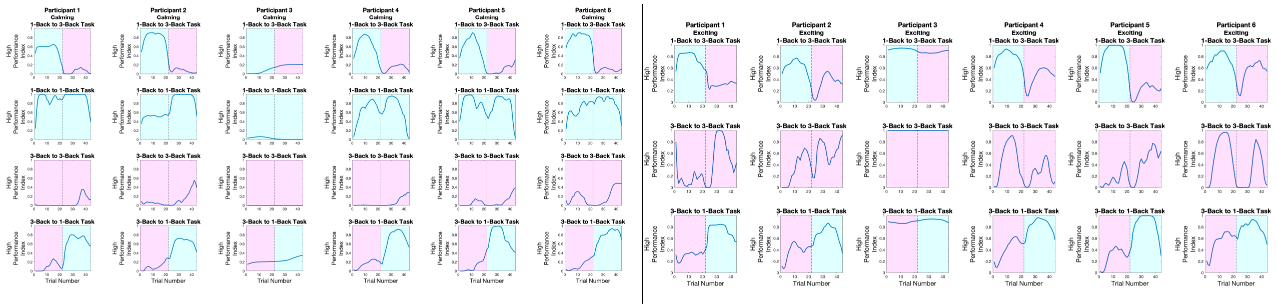

Fig. 8. **The transition of High Performance Index between different types of tasks.** The top sub-figures in the first four rows show how the average HPI changes from one type of task to another type of task during calming music for six participants. Similarly, the bottom sub-figures in the last 4 rows show how the average HPI changes from one type of task to another type of task during exciting music. The cyan and pink backgrounds correspond to the 1-Back and 3-Back Task.

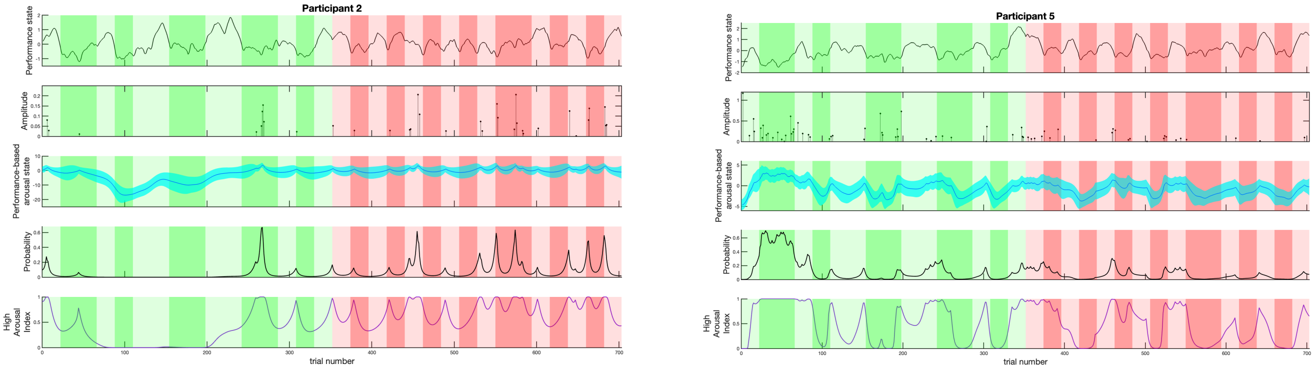

Fig. 9. **Arousal state estimation by performance-based filter for two participants.** The sub-panels for the figure respectively depict: the performance state signal (z_k); the average of the deconvolved neural impulses during trials ($\tilde{r}_k$); the estimated state ($\tilde{x}_k$) and its 95% confidence limits; the probability of impulse occurrence (ϕ_k); high arousal index. The background colors in each sub-panel depict: the 1-back task during calming session (light green); the 3-back task during calming session (dark green); the 1-back task during exciting session (light red); the 3-back task during exciting session (dark red).
